# Canine Serology as Adjunct to Human Lyme Disease Surveillance

**DOI:** 10.3201/1709.110210

**Published:** 2011-09

**Authors:** Paul Mead, Rohan Goel, Kiersten Kugeler

**Affiliations:** Author affiliation: Centers for Disease Control and Prevention, Fort Collins, Colorado, USA

**Keywords:** Lyme disease, zoonoses, Borrelia burgdorferi, Borrelia spp., serology, bacteria, dog, human, surveillance, seroprevalence, vector-borne infections, United States, dispatch

## Abstract

To better define areas of human Lyme disease risk, we compared US surveillance data with published data on the seroprevalence of *Borrelia burgdorferi* antibodies among domestic dogs. Canine seroprevalence >5% was a sensitive but nonspecific marker of human risk, whereas seroprevalence <1% was associated with minimal risk for human infection.

Lyme disease is caused by *Borrelia burgdorferi* and transmitted in North America by *Ixodes* spp. ticks. Routine surveillance for human illness indicates that risk for infection within the United States is highly localized. Residents of 10 states accounted for >93% of the ≈248,000 cases reported to the Centers for Disease Control and Prevention (CDC) during 1992–2006 (*1*). Annual county-level incidence ranged from 0 to >1,000 cases per 100,000 population (*1*).

Accurate information about risk is necessary for targeting and motivating Lyme disease prevention efforts (*2*). In addition, health care providers require knowledge of local disease risk to properly interpret clinical and laboratory findings (*3**,**4*). Although risk often can be inferred from surveillance data, reporting practices are subject to bias. Independent measures of disease risk are therefore valuable for validating surveillance findings.

Like humans, domestic dogs are susceptible to opportunistic infection with *B. burgdorferi*. These infections are often subclinical and pose no risk for direct transmission to humans. Nevertheless, they elicit a robust antibody response. Given the greater proclivity of dogs for tick exposure, canine seroprevalence has been proposed as a sensitive and independent measure of human Lyme disease risk (*5**–**7*). We compared US national surveillance data on Lyme disease with recently published data on *B. burgdorferi* antibody seroprevalence in dogs (*8*) to determine the degree of concordance between these 2 measures of Lyme disease risk and to assess the potential for canine seroprevalence to predict areas of Lyme disease emergence among humans.

## The Study

State and territorial health departments report Lyme disease cases to CDC as part of the National Notifiable Diseases Surveillance System (*1*). Data on canine seroprevalence of *B. burgdorferi* antibodies were obtained from a 2009 publication by Bowman et al. that reported results for 982,336 dogs tested throughout the United States by using a commercial C6-based assay during 2001–2006 (*8*). We obtained state-specific seroprevalence from Table 1 of this publication and county-specific seroprevalence as categorical values (0%, 0.1%–0.5%, 0.51%–1%, 1.1%–5%, >5.1%) from Figure 2 of this publication after digital enlargement. We excluded counties too small for the value to be determined reliably. We calculated average annual human Lyme disease incidence for 2001–2006 and 2007–2009 using US Census Bureau population estimates for 2004 and 2008, respectively. To evaluate county-level emergence of Lyme disease among humans, we stratified counties by the mean observed annual incidence for all counties during 2001–2006 of 4.7 cases per 100,000 population. We defined an emergent county as a county in which incidence was below this value during 2001–2006 and above this value during 2007–2009.

**Figure 1:**
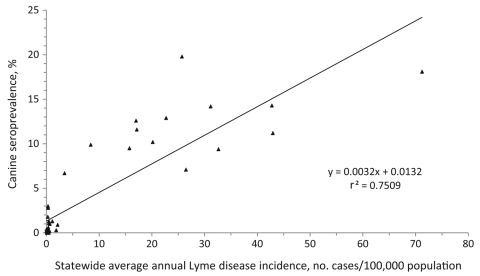
*Borrelia burgdorferi* antibody seroprevalence in dogs and reported Lyme disease incidence in humans, counties in 46 US states, 2001–2006.

Detailed canine seroprevalence data were available for 46 US states. In linear regression analysis, state canine seroprevalence and human Lyme disease incidence were positively correlated (Figure 1; r^2^ 0.75, p<0.001). On the basis of this relationship, human Lyme disease incidence was effectively zero when the canine seroprevalence was <1.3%. States generally fell into 2 distinct categories according to canine seroprevalence (Figure 1). Median Lyme disease incidence was uniformly low (median 0.3 cases/100,000 population) and not correlated with canine seroprevalence (r^2^ 0.0, p>0.4) among 32 states with canine seroprevalence <5%. Among 14 states with canine seroprevalence >5%, median annual human Lyme disease incidence was ≈100-fold higher (24.1 cases/100,000 population) and positively correlated with canine seroprevalence (r^2^ 0.33, p = 0.03).

**Figure 2:**
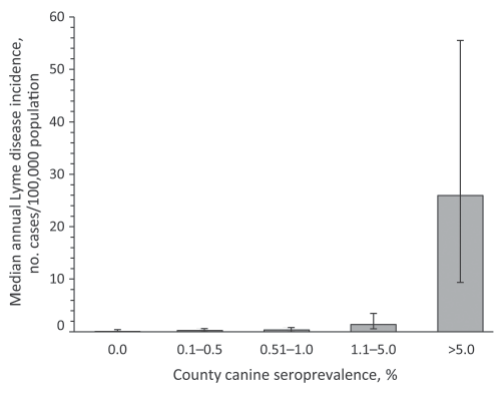
Median Lyme disease incidence in humans and *Borrelia burgdorferi* antibody seroprevalence in dogs in counties in 46 US states. Error bars represent 25th and 75th percentiles.

Categorical canine serologic data were available for 866 (28%) of 3,141 counties in the 46 states (*8*). Median population in 2004 was 85,699 for counties for which data were available, compared with 25,505 for all counties in the 46 states. As in the state-level analysis, human incidence and canine seroprevalence were positively associated at the county level. Median annual reported Lyme disease incidence for humans was 0.2 per 100,000 population in counties with canine seroprevalence <1%, 1.4 in counties with canine seroprevalence 1.1%–5%, and 25.9 in counties with canine seroprevalence >5% (p<0.001; Figure 2). Five (1%) of 520 counties with canine seroprevalence <1% had rates of human illness above the overall county mean of 4.7 cases per 100,000 population annually, compared with 171 (85%) of 201 counties with canine seroprevalence >5%.

Overall, 153 (5%) of 2,830 counties with average annual human incidence <4.7 per 100,000 population during 2001–2006 met the criteria for emergence during 2007–2009. Emergence was more common in counties with higher canine seroprevalence (Table). Eighteen (56%) of 32 counties with canine seroprevalence >5% met the criteria for emergence, compared with 6 (1%) of 519 counties with seropositivity <1% (p<0.001). Among the 32 counties with canine seroprevalence >5%, a total of 12 (67%) of the 18 counties with emergent Lyme disease were immediately adjacent to a county with seroprevalence >5%, compared with 4 (29%) of the 14 counties with nonemergent Lyme disease.

**Table:** Counties meeting criteria for emergence of human Lyme disease during 2007–2009, by canine seroprevalence of *Borrelia burgdorferi* antibodies during 2001–2006, 46 US states*

Canine seroprevalence, 2001–2006, %†	No. low-incidence counties,‡ 2001–2006	No. (%) emergent counties,§ 2007–2009¶
Unknown	2,065	96 (4.5)
0	240	1 (0.4)
0.1–0.5	174	1 (0.6)
0.51–1.0	101	4 (4.0)
1.1–5.0	122	33 (27.0)
>5.1	32	18 (56.3)

*All states except Alaska, Hawaii, Montana, and Nevada. †Data from Figure 2 in Bowman et al. (*8*). ‡Counties with below average incidence of human Lyme disease (<4.7 cases/100,000 population). §Counties with below average incidence in 2001–2006 and above in 2007–2009. ¶χ^2^ for trend 135.9, p<0.0001, for counties with known canine seroprevalence.

## Conclusions

Our results confirm an overall correlation between canine seroprevalence and reported human incidence of Lyme disease as measured through national surveillance. Canine seroprevalence <1% is associated with extremely low rates of human illness in both state- and county-level analyses. Because human cases are reported according to county of residence rather than county of exposure, infections acquired during travel will occasionally be reported from areas without local transmission. Similarly, low levels of canine seropositivity are expected on the basis of the specificity of assay (up to 2% false positivity [9]), data from field surveys (*7**,**10*), and relocation of dogs from areas of high endemicity (*8*). Low levels of canine seroprevalence or human incidence should not be misinterpreted as confirmation of local transmission of *B. burgdorferi*. Conversely, the overall agreement between human and canine data support the conclusion that risk for *B. burgdorferi* infection is generally low to nonexistent outside the highly Lyme disease–endemic areas of the Northeast, mid-Atlantic, and upper Midwest.

At the other end of the spectrum, canine seroprevalence >5% was invariably associated with above average Lyme disease incidence in state-level analyses. In county-level analyses, the situation was more nuanced. Although 85% of counties with canine seroprevalence >5% also had above average Lyme disease incidence, 15% did not. In more than half of these counties, incidence increased to above average rates in the following 3 years, suggesting some predictive potential for high canine seroprevalence, especially in counties geographically clustered with other high seroprevalence counties. In other counties, however, high seroprevalence appears to be an anomaly resulting from small sample sizes and local demographics. For example, Routt County, Colorado, is a small rural county in a state where locally acquired Lyme disease has never been documented. Although canine seroprevalence for the county was >5%, a survey of all county veterinarians indicated that 11 of 12 seropositive dogs had lived in or traveled to known Lyme disease–endemic areas (CDC, unpub. data). Selective testing of dogs with exposure histories may yield misleading results with respect to local endemicity.

Our findings suggest that canine seroprevalence >5% can be a sensitive but nonspecific marker of increased risk for human Lyme disease. Because dogs do not transmit infection directly to humans (or humans to dogs), this association reflects similar susceptibilities to tick-borne infection. In some circumstances, high canine seroprevalence appears to anticipate increasing rates of human infection at the county level. Conversely, canine seroprevalence <1% is associated with little to no local risk for human infection. Canine seroprevalence is a useful adjunct to human surveillance for Lyme disease.
